# Non-thyroidal Illness Syndrome in an Infant With Acute Anorexia and Psychological Stress

**DOI:** 10.7759/cureus.39803

**Published:** 2023-05-31

**Authors:** Masazumi Miyahara, Kyoko Osaki, Yukihiro Hasegawa

**Affiliations:** 1 Department of Pediatrics, Okanami General Hospital, Iga, JPN; 2 Division of Endocrinology and Metabolism, Tokyo Metropolitan Children's Medical Center, Tokyo, JPN

**Keywords:** psychologic stress, paralytic ileus, non-thyroidal illness syndrome, infant, hypothyroidism

## Abstract

Non-thyroidal illness syndrome (NTIS), a remarkable ensemble of changes in serum thyroid hormone concentration during acute illness, was first reported in the 1970s. While NTIS is not a form of hypothyroidism, it is characterized by a decrease in serum triiodothyronine (T3) or thyroxine (T4) or both with normal or decreased thyroid-stimulating hormone (TSH). Notably, it typically resolves without thyroid hormone replacement therapy. We report a case of paralytic ileus caused by NTIS in an infant with psychological stress. This case illustrates the development of NTIS during psychological stress, which can lead to severe symptoms such as those seen in pathological hypothyroidism.

## Introduction

Non-thyroidal illness syndrome (NTIS), also known as a euthyroid sick syndrome, refers to a transient decrease in serum triiodothyronine (T3) or thyroxine (T4), or both, while thyroid stimulating hormone (TSH) levels remain normal or decreased. NTIS is associated with various conditions, typically those that are acute and organic diseases, such as sepsis and myocardial infarction [1]; it is commonly considered a physiological adaptation rather than a pathological form of hypothyroidism. Thyroid hormone replacement is generally not required in patients with NTIS [2]. However, this condition may cause pathological responses, such as suppressed myocardial function, hypothermia, or impaired consciousness [3]. The pathophysiology of NTIS has not been completely elucidated to date. We report a case of an infant with paralytic ileus related to NTIS that may have developed in association with psychological stress.

## Case presentation

A one-year and six-month-old male patient presented with loss of appetite. Three days before admission, he had experienced decreased appetite, had drunk only a little water, and become sluggish. On admission, he notably had no fever, vomiting, or diarrhea. Before his anorexia developed, he had been able to eat only rice and a limited variety of boiled foods. He had also begun attending nursery school one month before symptom development. Moreover, the patient’s restricted food preferences and delay in language acquisition had led his primary care physician to suspect autism spectrum disorder. The patient had been delivered at 39 weeks and four days of gestation to a healthy mother with an uneventful pregnancy and had weighed 3090 g at birth. Newborn screening tests, including a test for TSH level, returned negative. The patient’s medical history and that of his family were unremarkable.

At admission four days after symptom onset, the patient was alert and had a temperature of 36.7 °C, heart rate of 135 beats/minute, and blood pressure of 110/70 mmHg. A physical examination revealed no remarkable findings; his height and weight were 76.0 cm [-1.61 standard deviation (SD)] and 9.0 kg (-1.25 SD), respectively. Laboratory analysis results were as follows - white blood cell count: 26,700 cells/µL (76% neutrophils); C-reactive protein (CRP): 0.32 mg/dL; aspartate aminotransferase (AST): 65 IU/L; alanine aminotransferase (ALT): 33 IU/L; creatine phosphokinase: 107 mg/dL; blood urea nitrogen (BUN): 23.3 mg/dL; creatinine: 0.23 mg/dL; sodium: 134 mEq/L; potassium: 5.4 mEq/L; chloride: 100 mEq/L; calcium: 10.1 mg/dL; phosphorus: 5.5 mg/dL; total cholesterol: 169 mg/dL; and glucose: 56 mg/dL. Additionally, venous blood gas analysis demonstrated a pH of 7.246, pCO_2_ of 23.1 mmHg, HCO_3_ of 9.8 mmHg, and base excess of -15.5 mmol/L (Table 1).

**Table 1:** Laboratory tests data on admission and three days after admission WBC: white blood cell count; RBC: red blood cell count; ALP: alkaline phosphatase; AST: aspartate aminotransferase; ALT: alanine aminotransferase; LDH: lactate dehydrogenase; CPK: creatine phosphokinase; T-Cho: total cholesterol; BUN: blood urea nitrogen; Crea: creatinine; CRP: C-reactive protein; pH: potential hydrogen; pCO_2_: carbon dioxide partial pressure; HCO_3_: bicarbonate; BE: base excess

Laboratory parameters	Patient value on admission	Patient value post-admission day 3	Reference range
Peripheral blood test			
WBC	26700/μL	12500/μL	4000-9000
Neutrophils	76%	50%	39-81
Lymphocytes	20%	48%	16-50
Monocytes	4%	2%	2-10
RBC	431 × 10^4^/μL	447 × 10^4^/μL	400-520 × 10^4^
Hemoglobin	11.9 g/dL	12.3 g/dL	13.0-17.0
Hematocrit	37.7%	36.9%	38.0-49.0
Platelet	56.9 × 10^4^/μL	47.9 × 10^4^/μL	12.0-44.0 × 10^4^
Serum biochemical test			
Total protein	6.9 g/dL		6.5-8.5
Albumin	4.7 g/dL		4.1-5.3
Total bilirubin	0.8 mg/dL		0.2-1.2
Glucose	56 mg/dL	74 mg/dL	70-109
ALP	744.0 U/L		138.3-468.7
AST	65 IU/L		10-35
ALT	33 IU/L		10-35
LDH	333 U/L		110-225
CPK	107 IU/L		50-200
T-Cho	169 mg/dl		150-219
BUN	23.3 mg/dL	10.1 mg/dL	9.0-22.0
Crea	0.23 mg/dL	0.19 mg/dL	0.50-1.10
Sodium	134 mEq/L	137 mEq/L	138-145
Potassium	5.4 mEq/L	4.9 mEq/L	3.4-4.7
Chloride	100 mEq/L	98 mEq/L	99-108
Calcium	10.1 mg/dL		8.5-10.5
Phosphorus	5.5 mg/dL		2.7-4.6
CRP	0.32 mg/dL		0.00-0.30
Venous blood gas test			
pH	7.246 mmHg	7.398 mmHg	7.320-7.410
pCO_2_	23.1 mmHg	40.5 mmHg	41.0-51.0
HCO_3_	9.8 mmHg	24.4 mmHg	24.0-28.0
BE	-15.5 mmol/L	-0.4 mmol/L	±3

**Figure 1:**
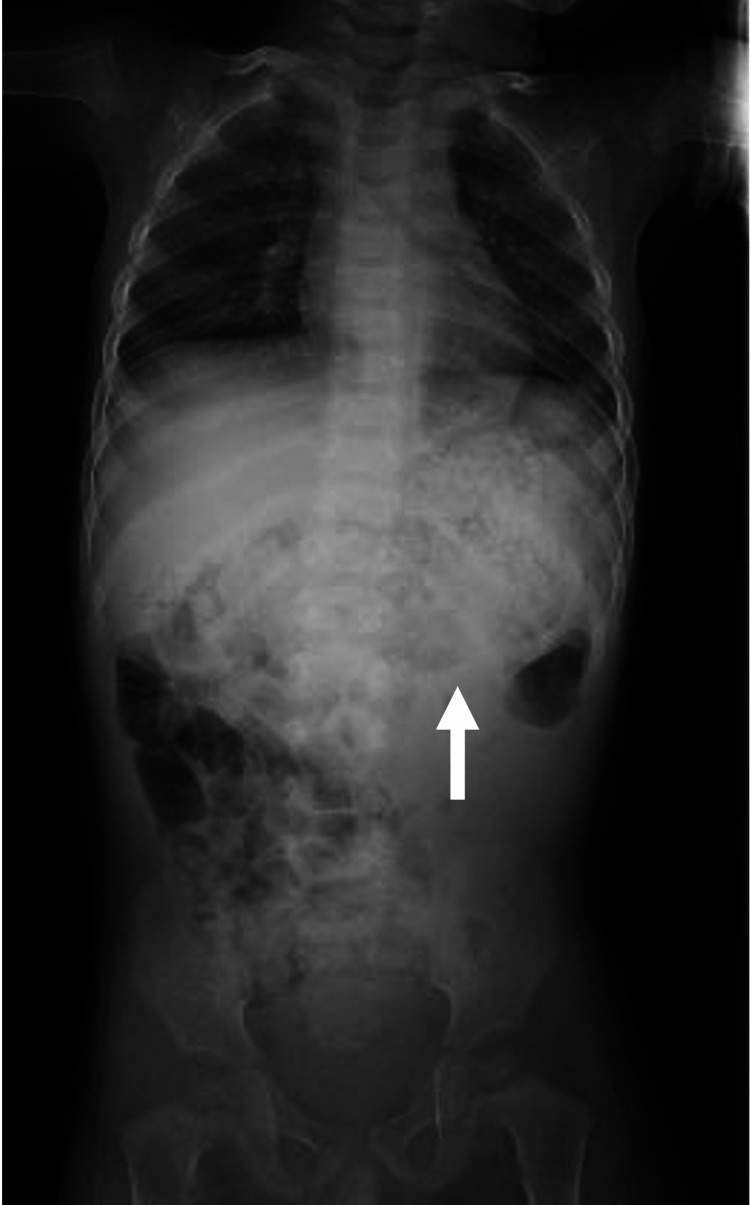
Abdominal X-ray of the patient at age 1 year and 6 months The white arrow shows the dilated stomach

Subsequently, intravenous fluid therapy was administered. Abdominal radiography revealed a dilated stomach with a large amount of content (Figure 1). Abdominal CT also demonstrated a dilated stomach and retention of contents but no abnormalities to account for the intestinal obstruction. Neither imaging test revealed a significant amount of stool retention. The imaging findings indicated paralytic ileus. Despite improvement in the metabolic acidosis and blood glucose after fluid therapy (venous blood gas analysis demonstrated pH of 7.398, pCO_2_ of 40.5 mmHg, HCO_3_ of 24.4 mmHg, base excess of -0.4 mmol/L, and blood glucose of 74 mg/dL on day three after admission) (Table 1), the patient did not consume food orally for four days after admission. Thyroid-related paralytic ileus was suspected, and a thyroid function test four days after admission revealed TSH of 0.06 μIU/mL, free T3 of <1.5 pg/mL, and free T4 of 0.68 ng/dL, indicating NTIS. MRI of the brain demonstrated no abnormalities of the pituitary gland or other areas.

The patient began to consume food, albeit in small amounts, from day five after admission and was discharged the next day. His thyroid function normalized without thyroid hormone supplementation; his TSH, free T3, and free T4 were 1.88 μIU/mL, 3.63 pg/mL, and 1.07 ng/dL, respectively, on day three after discharge when the patient’s appetite had normalized. In addition, the value of insulin-like growth factor-1 (IGF-1), adrenocorticotropic hormone (ACTH), and cortisol was 32 ng/mL, 22.1 pg/mL, and 15.4 μg/dL, respectively (Table 2). All these values were measured simultaneously and were normal. Based on these findings, NTIS was diagnosed as the cause of the patient’s abnormal thyroid hormone level. 

**Table 2:** Endocrinological test data TSH: thyroid stimulating hormone; T3: triiodothyronine; T4, thyroxine; IGF-1: insulin-like growth factor-1; ACTH: adrenocorticotropic hormone

Laboratory parameters	Patient value post-admission day 4	Patient value post-discharge day 3	Reference range
Endocrinological test			
TSH	0.06 μIU/L	1.88 μIU/L	0.35-4.94
Free T3	<1.50 pg/mL	3.63 pg/mL	1.68-3.67
Free T4	0.68 ng/dL	1.07 ng/dL	0.70-1.48
IGF-1		32 ng/mL	14-148
ACTH		22.0 pg/mL	7.2-63.3
Cortisol		15.4 μg/dL	4.5-21.1

## Discussion

NTIS, which occurs in response to various illnesses and fasting, is characterized by thyroid hormone inactivation with low T3 followed by TSH suppression. NTIS may be diagnosed in up to 70% of critically ill patients of all ages, including preterm neonates, term infants, children, and adults [4]. The question as to whether NTIS represents a beneficial, protective, and appropriate adaptive response to stress, critical illness, and malnutrition or whether it should be considered a harmful response to illness that requires correction remains unanswered. 

Our patient’s abnormal thyroid function was compatible with a diagnosis of NTIS. His thyroid function normalized after a short period without thyroid hormone replacement therapy; moreover, ACTH and GH deficiency were ruled out. However, no organic etiology of NTIS was identifiable during the patient’s clinical course.

There are two possible causes of NTIS in our case: psychological stress and anorexia. NTIS can be caused not only by physiological disorders but also by psychiatric disorders, such as depression [5]. Our patient was suspected of having autism spectrum disorder. The nursery school he had attended forced him to eat foods other than those he normally consumed, which had been likely stressful for the patient. The resulting psychological stress may have triggered the onset of NTIS. However, to the best of our knowledge, there are no reports of NTIS caused by psychological stress, and mental stress levels cannot be evaluated objectively. Hence, it cannot confidently be concluded that psychological stress caused NTIS in our patient. Anorexia is the other possible cause of NTIS. The patient presented with sudden anorexia. Prolonged anorexia and fasting can lead to NTIS [6]. As thyroid hormone abnormalities in NTIS often develop within 24-48 hours after the onset of starvation [7], his anorexia may have caused NTIS.

As far as paralytic ileus with anorexia is concerned, the low thyroid hormone levels in NTIS may have been a cause of the paralytic ileus. Paralytic ileus is a rare but well-known manifestation of hypothyroidism. There were no other, apparent abnormalities that may have caused the paralytic ileus, such as an electrolyte imbalance, drug effect, gastrointestinal infection (including peritonitis), shock, trauma, vascular occlusion, adrenal insufficiency, myotonic dystrophy, or renal failure. Radiography demonstrated a large amount of gastric content, indicating that, despite the patient’s having had a healthy appetite previously, he began experiencing paralytic ileus. 

The patient’s clinical course demonstrated that NTIS can cause severe symptoms, such as those observed in pathological hypothyroidism. Fortunately, the patient recovered quickly with symptomatic treatment. It is still debated whether or not thyroid hormone replacement therapy is required or is harmful in patients with NTIS [8]. One recent review stated that NTIS appears to be caused predominantly by increased peripheral inactivation of thyroid hormones, a beneficial adaptation of the body for reducing the expenditure of energy and activating the innate immune response [4]. However, in more severely and chronically ill patients, central suppression of the thyroid hormone axis undergoes pronounced changes, further accentuating the NTIS phenotype. This type of central suppression may not be adaptive and may require thyroid hormone replacement therapy.

Among pediatric patients, NTIS is frequently associated with cardiac surgery for congenital heart disease [8]. Nine interventional randomized clinical trials (IRCTs) enrolling a total of 364 children with NTIS after surgery for a congenital heart disease tested the efficacy of thyroid hormone supplementation. Although none of the IRCTs reported any clear effect on mortality, one trial found that T3 infusion improved myocardial function and reduced the need for postoperative intensive care [8,9]. Therefore, thyroid hormone replacement therapy may be effective at least in some patients with NTIS. The decreased free T3 and free T4 levels as well as the markedly decreased TSH level indicate that our patient had a NTIS phenotype with central suppression according to the terminology of Langouche et al. [4]. If the paralytic ileus observed in the patient had been prolonged or deteriorated, thyroid hormone replacement therapy would have been considered.

The limitations of this study include the inability to determine the cause-and-effect relationship with respect to anorexia, psychological stress, and NTIS. Psychological stress and anorexia are unquantifiable. However, the paralytic ileus was found while searching for the cause of anorexia and led to the detection of abnormal thyroid function. These thyroid function test results were entirely compatible with NTIS.

## Conclusions

The anorexia and psychological stress, which were clearly present in our patient, were presumably related to the concurrent NTIS. Although NTIS should be considered in any patients with acute or chronic organic conditions, it may be triggered by psychological stress. In addition, this case demonstrates that NTIS can cause serious symptoms such as paralytic ileus, similar to patients with true hypothyroidism.
